# Cultural differences in geographic mobility readiness among business management students in Germany and Spain ahead of graduation

**DOI:** 10.1007/s43545-021-00171-0

**Published:** 2021-06-25

**Authors:** Martin Mabunda Baluku, Janna Groh, Claudia Dalbert, Kathleen Otto

**Affiliations:** 1 Faculty of Psychology, Work and Organizational Psychology, Philipps University of Marburg, Gutenbergstraße 18, 35032 Marburg, Germany; 2 Martin Luther University of Halle-Wittenberg, Halle, Germany; 3 International Labour Organization, Geneva, Switzerland; 4 Makerere University, Kampala, Uganda

**Keywords:** Cultural differences, Geographic mobility, Individualism-collectivism, Uncertainty tolerance, Subjective norms

## Abstract

Given the increased internationalization of organizations and economies of scale concentrated in urban centers, graduates are often expected to relocate for their first job. Based on Hofstede’s model and Theory of Planned Behavior (TPB), we examine the effects of cultural dimensions (individualism-collectivism and uncertainty tolerance) as well as subjective norms (parents’ and peers’ attitudes towards geographic mobility) on readiness for geographic job-related mobility in samples of German and Spanish business management students ahead of graduation from university. The study involved administering a survey questionnaire to 273 third- and fourth-year business management students of two large universities (one in Germany and another in Spain). Cross-cultural measurement invariance of the measures was confirmed, allowing for comparison of scores across the groups. We found that German students had generally a larger geographical mobility, whose readiness was predicted by parents’ and peers’ perceived attitude and uncertainty tolerance. Readiness for geographic mobility was also higher when social or material incentives are offered, yet geographic mobility readiness for career incentives and for social incentives was predicted by vertical individualism and horizontal collectivism, respectively. This study is one of the first to examine geographic mobility readiness among undergraduate management students in the two countries, who by nature of their training are expected to be mobile. The study also shows the differential effects of sub-dimensions of the Hofstede cultural dimensions.

**Supplementary Information:**

The online version contains supplementary material available at 10.1007/s43545-021-00171-0.

## Introduction

Geographic mobility seems to be more of a necessity than ever for today’s workforce, even more so because labor migration has been significantly affected by the current coronavirus pandemic (Ivakhnyuk 2020). Moreover, with increased capacity to work virtually, the nature of expatriate work may change forever. Nonetheless, globalization and intercountry interactions are essential for development, and therefore are only temporary halted (Zimmermann et al. 2020). Before the pandemic, the heightened internationalization of companies’ operations had increased the demand for internationally mobile employees (Remhof et al. 2014). The earlier times slogan "today here, tomorrow there" was a sign of privilege, almost exclusively reserved for those of high potential (Baluku et al. 2019) seems to apply again given the current stringent migratory rules particularly due to the current coronavirus pandemic. However, international mobility had gain importance for career development (Baluku et al. 2019) before the pandemic. Whereas geographic career mobility may not gain the same prominence in the aftermath of the pandemic (Gamlen 2020), it will still be important especially to professionals in the field of business management, many of whom aspire to careers in international firms.

Professionals seeking to improve their career prospects within a company may also be expected to relocate, and they are often offered higher positions and better salaries as rewards for their mobility (Brett and Stroh 1997) in particular at early career stages (Lam et al. 2012). However, the readiness for an actual geographic mobility is relatively low, even in management positions (e.g. Collings et al. 2007; Selmer 2017). This raises a number of questions which we try to shed light on in this paper: In what respects do those who are willing to relocate for job reasons differ from those who are not? Under which circumstances are people willing to relocate? And, finally, are there cultural differences in (explaining) geographic mobility?

The significance of geographic mobility needs to be analyzed in a broad economic and sociological context. In a labor market marked by increasing globalization and competition, a growing pace of change and an increasing need for adaptation, geographic career mobility is part of the labor demand and supply ecosystem (Baruch and Altman 2016), particularly mobility regulates the demand and supply of labor therefore important for the organization of labor (Hao and Liang 2016). Given the increasing demand for mobile employees, it is important to learn more about the various personal reasons for or against mobility. People may move for the sake of their families or to improve their standard of living; their decisions may be motivated by social, private, or financial reasons. However, a substantial number of migrants move for career-related reasons (Naudé 2008; Roman and Paraschiv 2019). Earlier research shows that job relocation is considered an essential component of the economic and social life of many international firms and their employees (Crinò 2009; Daly and Geyer 1994; Munton and Forster 1990). While some employees would only consider a move to stay in work or to obtain employment, others would be willing to relocate for career-related incentives, such as a promotion, a company car, a pay rise, or generally prospects of career and personal advancement (for determinants of mobility, see: Baruch et al. 2016; Harpaz et al. 2002; Landau et al. 1992; Li and Walder 2001). However, it seems that the globalization era that espouses geographical career mobility is coming to an end or at best will be significantly halted by the coronavirus pandemic (Gamlen 2020). Yet the pandemic has evoked an unemployment crisis in some places (Blustein et al. 2020; Blustein and Guarino 2020; McGann et al. 2020) that may kindle a new wave of geographical career mobility in the aftermath. This paper analyzes the conditions under which people would be prepared to move, and perhaps even leave their country voluntarily, for job-related reasons.

### Geographic mobility readiness

A distinction needs to be made between job relocation and *geographic mobility readiness* for job-related reasons. Job relocation is defined as a complete act; i.e., an actual move to another city or country. Several studies have already investigated the determinants of this behavior (e.g. Brett and Reilly 1988; Kornblum et al. 2018; McCollum et al. 2018; Ng et al. 2007). The geographic mobility readiness, in contrast, reflects attitudes toward geographic mobility and hence predicting possible future moves since attitude is a predictor of behavioral intention and actual behavior (Ajzen 1991); and is a predictor of success in expatriate work (Weisheit 2018). Unsurprisingly, geographic mobility readiness is an important predictor of job relocation decisions and behavior, as several studies have shown (e.g. Brett and Reilly 1988; Cotton and Majchrzak 1990; Weisheit 2018).

Sociological research further differentiates according to the duration of a potential move (Schneider 2002). An employee might be geographically mobile in the short term, when a contract involves traveling or working away on a construction job (Kathleen Otto and Dalbert 2010), for example. In such cases, mobility is only a temporary requirement; a permanent change of residence is not necessary. Thus, the geographic mobility readiness includes consideration of whether the move is short-term or long-term relocation and expatriation.

Over the years, researchers have investigated why individuals move and determinants of job-related geographic mobility. Nonetheless, with few exceptions (e.g. Baluku et al. 2018; Dette and Dalbert 2005; Netz and Jaksztat 2014; Kathleen Otto and Dalbert 2012), most studies have investigated employees or professionals, respectively (Andresen and Margenfeld 2015; Eby and Russell 2000; Kornblum et al. 2018; Kathleen Otto and Dalbert 2012). The present study, in contrast, analyzes the readiness of students who are nearing the end of their university studies and facing the challenge of finding their first job to leave their university town. University graduates are ideally suited to mobility: most of them are young, unmarried, and childless, and their intellectual level is comparably high (List 1996). To ensure the best fit between their qualifications and their first job, graduates must be ready to move. This should apply especially to business management students, many of whom aspire to careers in international firms, and sometimes already decide to study abroad (Baluku et al. 2019; Presley et al. 2010). To sum up, the present study examines the willingness of business management students to be geographically mobile for job-related reasons.

### Theory and hypothesis development

Readiness for geographical job mobility mirrors the intention of an individual to work in places other than one’s hometown or country, hence can be studied from the behavior intentions perspective (Ajzen 1985, 1991). Intentions refer to the readiness to engage in a given behavior (Ajzen 2011), and are considered the best predictor behavior (Van Gelderen et al. 2015). The theory proposes that behavioral intentions are a function of attitudes toward the given behavior, subjective norms, and perceptions of behavioral control (Ajzen 1991, 2002). The present study emphasizes culture and subjective norms as predictors of readiness to be geographically mobile. Previous research has found that the antecedents of behavior intentions specified in the TPB are underlying mechanisms for linking personal variables such as personality and the intention to work abroad (Remhof et al. 2014). The present study focuses on specific subjective norms; namely perceived parents’ and peers’ attitudes towards geographic mobility. We further propose that there are cultural differences in the readiness for geographic job-related mobility. Based on the Hofstede model (Hofstede et al. 2010; Hofstede and Bond 1984). Hofstede's (1984) initial model comprised four dimensions. However, the model has undergone several modifications and currently consists of six dimensions: power distance, uncertainty avoidance, individualism versus collectivism, masculinity versus femininity, long versus short term orientation, and indulgence versus restraint (Hofstede 2011). These dimensions precisely connote how societies respond to basic social issues (Minkov and Hofstede 2011) therefore provide a framework for understanding why individuals from a given society behave or respond to stimuli in specific patterns. This includes individuals’ career mobility preferences. For the present study, we particularly examine the effect of individualism-collectivism and uncertainty tolerance dimensions. A detailed discussion of the role of cultural differences in career and geographic mobility is made in the subsequent section.

#### Cultural differences

The technologically developed countries differ widely in terms of the frequency of job relocation. Earlier evidence suggests, for example, that the percentage of household moves per year is much higher in the United States of America (USA) and Canada than it is in Europe (Clark and Drever 2000; Gober 1993). In the Anglo-American countries, higher acceptance of geographic mobility and less protection against layoffs has led to a “culture of migration” (Gober 1993). The present study concerns geographical mobility in two European countries. Whereas the cooperation among European Union member states is becoming increasingly important in facilitating career-related geographic mobility, the mobility readiness differs from one state to the next due to variations in cultures and economic situations (Diprete et al. 1997; Teichler 2015). Consequently, there are also differences in the benefits of mobility among countries (Storz et al. 2015). Cultural differences in aspects such as the meaning of social networks or the acceptance of uncertainty may also affect geographic mobility. Over the past twenty years, these two psychological dimensions have been analyzed on a personal and a cultural level (Hofstede 1980; Hofstede et al. 2010; Minkov and Hofstede 2011).

In the present paper, we focus on the cultural perspective and compare the readiness of business management students in two European capitals, Berlin and Madrid, to relocate after graduation. In the Hofstede model, particularly cultural differences between the Germans and the Spanish can be expected on two cultural dimensions: uncertainty avoidance and individualism (Hofstede 1980, 2001; Hofstede et al. 2010). Increasing individualization, i.e., liberation from traditional structures, resulting in more personal freedom of choice, has been a matter of discussion since the 1960s (Herriot and Scott-Jackson 2002; Lukes 1969). It is sometimes perceived as a danger to established social structures, as it entails more uncertainty, a less social orientation, and hence lower social responsiveness (Mezzenzana 2020). Hofstede’s seminal work on culture compared Germany and Spain on both dimensions and revealed that scores lower on individualism and higher on uncertainty avoidance than Germany (Hofstede 1980, 2001; Hofstede et al. 2010). This might have consequences for geographic mobility; given that people in individualistic cultures are more like to be mobile as they are more willing to change workgroups or locations (Sullivan and Arthur 2006). Similarly, individuals from cultures that are low in uncertainty avoidance are likely to embrace job-related mobility as they are less threatened by change.

#### Individual and cultural predictors of the geographic mobility readiness

The implications of social integration can be reflected on people’s social orientations, as conceptualized within individualism-collectivism research (Hui and Triandis 1986; Triandis et al. 1988). Collectivists value social integration, and thus the attitudes of the people around them, whereas individualists put their own goals above those of others. This categorization has been further differentiated by the concepts of vertical and horizontal alignment (Singelis et al. 1995; Triandis 1998), which are comparable to Hofstede’s dimension of power distance. Whereas vertical alignment involves clear status differences and competition between individuals, horizontal alignment symbolizes cooperation and few status differences. Thus, in horizontal individualism, the individual is autonomous, but not of higher or lower status than any other individual (Singelis et al. 1995). On the other hand, vertical individualism is characterized by autonomy as well as acceptance of inequality which is reflected in the existence of a clear hierarchical structure between individuals of different status (Singelis et al. 1995).

Similarly, collectivism also has horizontal and vertical sub-dimensions. Horizontal collectivism emphasizes equality, which is reflected by the importance placed on group membership and equal status among group members (Singelis et al. 1995; Triandis 1998). Whereas group membership is also important in vertical collectivism, there are clear status differences between members of a group. This implies that individuals can lose their social network especially if they move to a different geographical location. Moreover, losing social ties is especially difficult for collectivists. The high value placed on inter-group boundaries in collectivistic cultures (Rychlowska et al. 2015) makes migration difficult for individuals, including moving for job purposes. In this direction, previous studies conducted among high school students as well as apprentices about to enter the job market found that the less collectivist and the more individualist they were, the readier the young people were to be geographically mobile (Dette and Dalbert 2005; Kathleen Otto and Dalbert 2012).

The cultural context defines the social environment, either encouraging or discouraging geographic mobility. Particularly, the attitude of the social environment towards geographic mobility plays an important role. According to the theory of reasoned action (Martin Fishbein and Ajzen 2010), and its extension to the theory of planned behavior (Ajzen 1991), subjective norms, i.e., the way the social environment is thought to perceive a particular behavior, is an important determinant of the intention to execute that behavior. In the context of university students’ geographic mobility readiness, subjective norms are reflected by both their parents’ and peers’ attitudes to geographic mobility. These are part of the socialization processes for students, which influence work or career preferences (Rodrigues et al. 2013). Consequently, family issues tend to influence mobility readiness and behavior (Patton and Doherty 2020). Recent research has shown that perceived social attitudes to mobility have a notable influence on personal readiness to relocate or to work in a high-mobility job (Dette and Dalbert 2005; Eby and Russell 2000; Kathleen Otto and Dalbert 2010, 2012), as well as to take expatriation assignments (Seibert et al. 2001). Moreover, such family and parental influences tend to have greater impact where the culture is more oriented towards collectivism (Sawitri and Creed 2017). To summarize, it is likely that people who respect subjective norms and value their own integration in a social network will be less willing to be geographically mobile; suggesting that a more collectivist culture (*Spain*) are expected to score lower on geographic mobility than a more individualist culture (*Germany*).^1^ Although, there is evidence suggesting that expatriates preferring inclusion (collectivistic) in the host community are more integrated and adjusted to work (Ditchburn and Brook 2015).

Another cultural factor that has been shown to affect geographic mobility readiness and behavior is tolerance of ambiguity or uncertainty (Albrecht et al. 2018; Andresen and Margenfeld 2015; Brower et al. 2019; Dalbert 2002; Dette and Dalbert 2005). Ambiguity tolerance is associated with openness to experience and flexibility, which in turn predicts mobility and success in expatriation work (Baluku et al. 2018; Shaffer et al. 2006; Tarique and Weisbord 2013). This individually varying construct can be seen as similar to Hofstede's (1980) concept of uncertainty avoidance. Uncertainty avoidant cultures perceive a high level of stress when confronted with an unknown future (Hofstede 2001) which they try to overcome “by reliance on social norms, rituals, and bureaucratic practices” (House et al. 2002, p. 5). Uncertain situations, of which relocations are a prime example, are defined as ambiguous, complex, and difficult to manage (Stanley Budner 1962). People differ in their ability to cope with such situations (Dalbert 2002). Those high in uncertainty tolerance see such a situation as a challenge, whereas those low in uncertainty tolerance consider them as a threat and try to avoid them. To sum up, people lower in uncertainty tolerance are expected to be less ready to relocate (Kathleen Otto and Dalbert 2012), yet uncertainty tolerance is higher in Germany than in Spain^2^ (Wennekers et al. 2010).

Overall, we expected the business management students in our study to report higher geographic mobility readiness: (H1) the more uncertainty tolerant they are *(H1)*; the more individualistic *(H2a*) and less collectivistic they are (*H2b*); the more positive they perceive their parents’ (*H3a*) and peers’ (*H3b*) attitudes to geographic mobility. Furthermore, German students were expected to be: more uncertainty tolerant (*H4a*); individualistic (*H4b*); and less collectivistic *(H4c)* than the Spanish students. Consequently, we expected the German students to be more willing to relocate than Spanish students *(H5)*.

## Methodology

To investigate the present hypotheses, a questionnaire study was administered to university students one to two years before graduation. We chose a sample of business management students, for whom geographic job-related mobility is highly likely, especially if they want to work in global organizations.

### Sampling criteria and data collection process

As we planned to conduct a cross-cultural comparison specifically between the two countries Germany and Spain all business management students should be of German or Spanish origin. Moreover, as geographic mobility decisions very much depend on one’s familial situation, we focused on students who are (still) childless and who are younger than 30 years of age to guarantee that the personal conditions across the sample is somewhat similar.

Data collection took place at universities of the two capitals, i.e. at the Universidad Autónoma de Madrid and the Freie Universität Berlin. To explore students ahead of graduation only third- and fourth-year students were invited to participate during their lectures. Lecturers remained in class during the assessment. Although participation was voluntary, all students took part.

Overall a total of 273 students participated and filled in the questionnaires. Of these those who had children, who were older than 30 years of age, who originated from other countries than Germany or Spain, or who did not complete the questionnaires were excluded from the analyzes leaving a sample of 222 business management students (40.6 percent male) with a mean age of *M* = 22.98 (*SD* = 2.06).

### Sample description

The resulting sample consisted of 108 Spanish students (39.3 percent male) and 114 German students (42.0 percent male). Details regarding the sample characteristics of the two countries can be found in Table 1.

**Table 1:** Sample characteristics of the Business Management Students by country

	German students (*N* = 114)	Spanish students (*N* = 108)
*Sex*		
Male	*n* = 47	*n* = 42
Female	*n* = 65	*n* = 65
Non-specified/Other	*n* = 2	*n* = 1
*Age*	*M* = 23.98 (*SD* = 1.98)	*M* = 21.94 (*SD* = 1.56)
*Students’ relationship status*		
Single/no relationship	*n* = 64	*n* = 59
In relationship/married	*n* = 47	*n* = 49
*Probability of finding job in university town*	*M* = 3.53 (*SD* = 1.45)	*M* = 4.69 (*SD* = 1.15)
*Number of prior relocations*	*M* = 2.10 (*SD* = 0.86)	*M* = 1.91 (*SD* = 0.82)
*Distances of prior relocations*	*M* = 1.84 (*SD* = 1.03)	*M* = 1.04 (*SD* = 1.00)

Probability of finding a job in one’s university town ranged from 1 (very unlikely) to 6 (very likely), and distances of prior relocations from 0 (no relocation) to 3 (relocation to another country). In cases where the numbers across the categories do not add up to 114 for German and 108 for Spanish students, some data are missing

The two groups were tested for demographic differences using χ^2^-statistics for categorical variables and t-tests for independent samples for continuous variables. No significant differences were found in terms of the gender ratio, χ^2^ = 0.17; *n.s.* (see, Table 1) or the students’ relationship status, χ^2^ = 0.20; *n.s.*, with some 42 percent of German students and 45 percent of Spanish students reporting being in a relationship.

The two samples did differ significantly, however, in terms of the students’ age, and the perceived probability of finding a job in the university town after graduation. On average, the Spanish students were two years younger than the German students, *t* = − 8.53; *p* < 0.001, and were more optimistic about finding a job in their place of residence, *t* = 6.55; *p* < 0.001. Finally, we assessed our participants’ prior experiences of mobility by asking them to specify the number *and* distances of prior relocations (0 “no relocation,” 1 “relocation within the university town,” 2 “relocation to another city,” and 3 “relocation to another country”). There was no significant difference in the number of prior relocations, *t* = − 1.68; *n.s.*, but the samples differed significantly when it comes to the distances of relocations. Relative to the Spanish students, the German students had higher mobility, measured in terms of international distances moved, *t* = − 5.86; *p* < 0.001.

### Research instruments

All measures were originally developed or already existed in German. The Spanish translation of the instruments was verified using independent back-translation (Brislin 1970). All items were given on a 6-point scale from 1 “strongly disagree” to 6 “strongly agree.” Scale means were used as variables. When more than one item per scale was missing, the whole scale was defined as missing.

#### Geographic mobility readiness

Taking a multi-method approach (Campbell and Fiske 1959), we measured the students’ geographic mobility readiness in different ways. General mobility readiness was assessed using the 10-item Geographic Mobility Scale (Dalbert and Otto 2004); e.g., “I would move to another city for a better job”), which taps the willingness to be geographically mobile for job-related reasons (German sample: α = 0.84; Spanish sample α = 0.83). To measure the geographic mobility readiness for certain incentives, we constructed a 27-item scale (see Appendix) based on three vignettes describing different types of job mobility. A principal component analysis with Varimax rotation identified three stable factors distinguishing three kinds of incentives: social incentives such as nice people, friends, or relatives living nearby (9 items; German sample: α = 0.82; Spanish sample α = 0.81), material incentives such as a company car or housing (6 items; German sample: α = 0.86; Spanish sample α = 0.85), and career incentives such as career prospects, professional development, a higher position or better salary (12 items; German sample: α = 0.95; Spanish sample α = 0.92).

#### Predictors of geographic mobility readiness

We assessed *uncertainty tolerance* using the 8-item Uncertainty Tolerance Scale (Dalbert 2002; e.g., “I like to try things out, even if nothing comes of it”) which has already been successfully applied in cross-cultural research (Kathleen Otto et al. 2011). One item was excluded because it showed a low item-total correlation in the Spanish sample (*r*_*it*_ = 0.10); a reduced 7-item scale was thus used in the analyzes (German sample: α = 0.78; Spanish sample α = 0.60).

We used the *individualism-collectivism* scales (Singelis et al. 1995), the German translation (Dalbert and Grob 2000), to assess the relative importance of friendships and family ties. Each of the four dimensions (horizontal individualism, vertical individualism, horizontal collectivism, and vertical collectivism) is measured by 8 items. Seven items had to be excluded because of low item-total correlations (*r*_*it*_ < 0.20) in one of the samples. Thus, the final horizontal individualism scale consisted of 5 items (German sample: α = 0.62; Spanish sample α = 0.61; e.g. “I like my privacy”), the vertical collectivism scale of 4 items (German sample: α = 0.54; Spanish sample α = 0.60; e.g., “I hate to disagree with others in my group”), and vertical individualism (German sample: α = 0.79; Spanish sample α = 0.75; e.g., “Winning is everything”) and horizontal collectivism scales (German sample: α = 0.67; Spanish sample α = 0.79; e.g., “I feel good when I cooperate with others”) of 8 items each.

Although the Cronbach’s αs indicated rather low internal consistencies, these findings are in line with Singelis et al. (1995) research, in which the reliability coefficients of several subscales were also below 0.70 (for similar findings see Otto and Dalbert 2012; Sivadas et al. 2008). As alpha is dependent on the length of a scale, and the breadth of the measure, it is important to also consider inter-item correlations particularly for short scales (Streiner 2003). Clark et al. (1995) suggested that mean inter-item correlations between 0.40 and 0.50 should be yielded for scales measuring very narrow characteristics and between 0.15 and 0.20 for scales measuring broad characteristics. This latter criterion was met by horizontal individualism (*r*_e__s__t_ > 0.24 for both countries), vertical collectivism (*r*_e__s__t_ > 0.23 for both countries), and horizontal collectivism (*r*_e__s__t_ = 0.20 for the German sample).

To tap *social norms*, we assessed the students’ perceptions of their parents’ and peers’ attitudes to geographic mobility with two parallel 4-item scales (parents’ attitude: German sample: α = 0.72; Spanish sample α = 0.86; e.g., “My parents would like it if I spent a year abroad”; peers’ attitude: German sample: α = 0.78; Spanish sample α = 0.77; e.g., “My friends would like to spend a year abroad”).

In addition to the country of origin (0 = Spain; 1 = Germany), three socio-demographic variables were included in the analyzes: gender (0 = male; 1 = female), age, and relationship status (0 = single; 1 = in relationship). To judge the need for job-related relocation in the near future, the expected graduation date (next year; in two years or later) and the subjective probability of finding a job in the university town after graduation (ranging from 1 “very improbable” to 6 “very probable”) were included. Finally, previous experiences of relocation were described in terms of the number and distances of prior relocations (0 = “no relocation,” 1 = “relocation within the university town,” 2 = “relocation to another city,” 3 = “relocation to another country”). In sum, eight control variables were included in the analyzes in addition to the seven personal dispositions.

### Testing for cross-cultural measurement invariance

Measurement invariance is important in cross-cultural studies to ascertain whether the instruments measured the same psychological constructs in the different cultures or countries. We, thus, compared if the factor structure of the variables in both the German and Spanish samples is equivalent. Such comparisons are however appropriate if at least partial measurement invariance is given (Byrne et al. 1989). To achieve this, we conducted six separate sets of two-group confirmatory factor analyzes computed using Amos (Arbuckle 2010). The items of each scale were specified to load only on their latent factor. In the first step of the analyzes, we followed an algorithm and checked our measures for the configural invariance. This was followed by metric invariance in a second step, and lastly, the scalar invariance in the final step (Milfont and Fischer 2010). These tests assessed whether the given elements (including factor loadings, item intercept, and factor variance) were equal across groups.

As indices to evaluate the overall model fit, we relied on the Chi-square-to-degrees of freedom ratio (χ^2^/*df*), and the root mean square error of approximation (RSMEA; Browne and Cudeck 1993). The χ^2^ difference as well as the comparative fit index (CFI; Satorra and Bentler 1994) were applied as incremental fit indices to estimate improvement over competing models.

## Results

### Mean differences

The results of the measurement invariance tests are provided in Table 2. Except for the geographic mobility readiness for incentives measure, at least full metric invariance (RSMEA < 0.08; CFI > 0.90) was observed. If this criterion is satisfied, ratings can be compared across groups (Milfont and Fischer 2010) allowing us to test our hypotheses.

**Table 2:** Fit indices for measurement invariance tests

Model	RMSEA						
	χ^2^	*Df*	χ^2^/*df*	Value	90%CI	CFI	Δχ^2^
*Uncertainty tolerance*							
Full configural invariance	48.64	34	1.43	.05	.00–.07	.95	–
Full metric invariance	62.70	40	1.57	.05	.02–.08	.92	14.06*
Full scalar invariance	111.87	47	2.38	.08	.06-.10	.76	49.17***
*Individualism*							
Full configural invariance	134.41	122	1.10	.02	.00–.04	.98	–
Full metric invariance	180.23	133	1.36	.04	.02–.06	.92	45.81***
Full scalar invariance	238.11	146	1.63	.05	.04–.07	.84	57.88***
*Collectivism*							
Full configural invariance	130.48	103	1.27	.04	.01–.05	.93	–
Full metric invariance	144.18	113	1.28	.04	.01–.05	.92	13.69
Full scalar invariance	261.70	125	2.09	.07	.06–.08	.63	117.52***
*Social norms*							
Full configural invariance	75.68	44	1.72	.06	.03–.08	.95	–
Full metric invariance	81.73	50	1.64	.05	.03–.07	.95	6.05
Full scalar invariance	102.65	58	1.78	.06	.04–.08	.93	20.92**
*General Geographic MR*							
Full configural invariance	114.29	67	1.71	.06	.04–.08	.93	–
Full metric invariance	119.50	76	1.57	.05	.03–.07	.94	5.21
Full scalar invariance	201.97	86	2.34	.08	.07–.09	.83	81.62***
*Mobility readiness incentives*							
Full configural invariance	1,375.23	528	2.61	.09	.08–.09	.83	–
Full metric invariance	1,424.67	551	2.59	.09	.08–.09	.82	49.44**
Full scalar invariance	1,554.80	578	2.69	.09	.09–.10	.80	130.13***

*RMSEA* Root mean square error of approximation, *CFI* Comparative fit index, *CI* Confidence interval, *MR* mobility readiness

*p < .05. **p < .01. ***p < .001

First, an analysis of variance (ANOVA) with the two between-subject factors country of origin and gender was run to test for differences in uncertainty tolerance, individualism-collectivism, and perceived attitudes of parents and peers (see, Table 3 for details). Following Cohen (1988), the effect sizes are reported for each analysis to give an overview of the significance of the results. The effect sizes can be interpreted in the following way: η_p_^2^ ≥ 0.02 corresponds to a small effect, η_p_^2^ ≥ 0.15 to a medium effect, and η_p_^2^ ≥ 0.35 as to a large effect.

**Table 3:** Intercorrelations of all scales separately for the German and Spanish samples, and mean differences across gender and cultures

													Germany		Spain		*F*
													Female	Male	Female	Male	
		1	2	3	4	5	6	7	8	9	10	11	*M* *(SD)*	*M* *(SD)*	*M* *(SD)*	*M* *(SD)*	
1	Uncertainty tolerance	–	− .14	− .02	− .02	− .13	− .17	− .07	− .27**	− .14	− .09	− .02	3.78 (0.84)	3.66 (0.71)	3.77 (0.63)	3.84 (0.59)	10.68
2	Horizontal individualism	− .29**	–	− .22*	− .07	− .07	− .11	− .08	− .07	− .04	− .05	− .03	4.50 (0.58)	4.65 (0.63)	4.47 (0.73)	4.57 (0.73)	10.74
3	Vertical individualism	− .14	− .19*	–	− .17	− .04	− .10	− .04	− .11	− .45**	− .06	− .14	3.50_ab_ (0.66)	3.97_c_ (0.91)	3.31_a_ (0.80)	3.66_bc_ (0.61)	11.80**
4	Horizontal collectivism	− .08	− .02	− .16	–	− .36**	− .07	− .14	− .11	− .16	− .23*	− .15	4.92_a_ (0.49)	4.80_a_ (0.53)	5.27_b_ (0.42)	4.85_a_ (0.68)	19.99**
5	Vertical collectivism	− .01	− .01	− .33**	− .12	–	− .05	− .09	− .10	− .05	− .04	− .14	3.37_a_ (0.84)	3.47_a_ (0.79)	4.07_b_ (0.66)	3.91_b_ (0.88)	11.10**
6	Parents’ attitude to GM	− .04	− .07	− .23*	− .10	− .06	–	− .38**	− .34**	− .37**	− .15	− .12	4.16_a_ (0.96)	4.46_b_ (0.90)	4.20_a_ (1.15)	4.64_b_ (0.91)	12.63
7	Peers’ attitude to GM	− .04	− .04	− .11	− .35**	− .16	− .38**	–	− .36**	− .23*	− .02	− .05	4.70 (0.77)	4.52 (1.04)	4.85 (0.75)	4.67 (0.93)	11.39
8	General Geographic MR	− .13	− .06	− .03	− .05	− .04	− .29**	− .26**	–	− .48**	− .07	− .12	4.11_a_ (0.86)	3.99_a_ (0.86)	3.71_b_ (0.86)	3.91_b_ (0.82)	12.46
9	MR Career incentives	− .04	− .13	− .29**	− .12	− .05	− .28**	− .14	− .40**	–	− .14	− .52**	4.39 (0.93)	4.72 (1.06)	4.46 (0.96)	4.62 (0.73)	11.38
10	MR Social incentives	− .01	− .08	− .06	− .26**	− .31**	− .14	− .17	− .12	− .20*	–	− .35**	3.61_a_ (0.97)	3.76_a_ (1.02)	4.38_b_ (0.99)	4.19_b_ (0.94)	18.38**
11	MR Material incentives	− .09	− .11	− .20*	− .12	− .09	− .17	− .02	− .07	− .29**	− .27**	–	3.29_a_ (1.22)	3.08_a_ (1.08)	3.61_b_ (1.07)	3.99_b_ (1.10)	15.71**

All scale values ranged from 1 to 6, with 6 indicating strong endorsement of the construct. GM = geographic mobility. MR = mobility readiness. For intercorrelations, the upper diagonal reflects the German sample, and the lower diagonal the Spanish sample. For ANOVA’s, subscripts with different letters indicate significant mean differences

^*^ *p* < .05; ** *p* < .01; *p* < .10 (two tailed tests)

Regarding *uncertainty tolerance*, no differences were found for gender, country of origin, or their interaction. For *horizontal individualism*, no significant differences were found in relation to gender and country of origin, or for their interaction. For *vertical collectivism*, only the main effect of country of origin (η_p_^2^ = 0.12) was significant. As hypothesized, we found German business management students to score lower on vertical collectivism than Spanish students. For *horizontal collectivism*, the main effects of both gender (η_p_^2^ = 0.07) and country of origin (η_p_^2^ = 0.03), as well as their interaction (η_p_^2^ = 0.02) did prove to be significant. Male German students scored lowest on horizontal collectivism, followed by male Spanish students and female German students – which all did not substantially differ from each other, while female Spanish students scored significantly higher on horizontal collectivism (see, Table 3). With respect to *vertical individualism*, the main effects of gender (η_p_^2^ = 0.10) and country of origin (η_p_^2^ = 0.05) were significant, but not the interaction effect. As expected, German students scored higher on vertical individualism than Spanish business management students, and males scored higher than females. More specifically, female Spanish students differed in their endorsement of vertical individualism from male students independent from their culture of origin, while female German students differed from male German students only. Concerning *parents’ attitudes to geographic mobility*, we found significant differences for gender (η_p_^2^ = 0.03) – with males perceiving their parents as being more strongly in favor of mobility than females – but no effects of country of origin or the interaction of gender and country of origin. Finally, for *peers’ attitudes to geographic mobility*, no substantial differences were found.

A multivariate analysis of variance (MANOVA) with mobility (i.e., the four dimensions of geographic mobility) as the within-subject factor and country of origin and gender as the two between-subject factors was performed to test for differences in the general geographic mobility readiness and the geographic mobility readiness for certain incentives. Wilk’s lambda indicated a significant within-subject effect (*F* = 82.29; *p* < 0.001; η_p_^2^ = 0.54). In addition, for the between-subject factors the two-way interaction of mobility with the country of origin (*F* = 9.41; *p* < 0.001; η_p_^2^ = 0.12) and the three-way interaction of mobility with the country of origin and gender (*F* = 4.46; *p* = 0.005; η_p_^2^ = 0.06) became significant. No significant main effects were found for gender. To determine the relevance of gender and country of origin for the mobility factor, an ANOVA with the two between-subject factors country of origin and gender was performed for each mobility dimension separately. In these further analyzes, we did not find any significant main effects for gender or for the interaction of gender and country of origin for any mobility dimension. However, as shown in Table 3, for general geographic mobility readiness (η_p_^2^ = 0.02), mobility readiness for social (η_p_^2^ = 0.09) as well as material incentives (η_p_^2^ = 0.07) – but not for mobility readiness for career incentives – a significant main effect of country of origin was observed. As expected, the German business management students were generally more ready to move than their Spanish counterparts, while in line with our reasoning, the Spanish business management students were more ready to be geographically mobile when social incentives were offered and/or when material incentives beckoned.

To summarize, in line with our fourth hypothesis, the German business management students scored higher on vertical individualism (H4b) and lower on vertical and horizontal collectivism (H4c) than the Spanish students. Furthermore, in line with our fifth hypothesis, the German students were generally more willing to be mobile after graduation, but the Spanish students were more responsive to social and material incentives. Note, however, with the exception of the MANOVA, our results only revealed small effects. In addition, the country groups did not differ in terms of uncertainty tolerance (which contradicts H4a), perceived attitudes of peers and parents, or responsiveness to career incentives.

### Explaining geographic mobility readiness: regression analyzes

Ordinary Least-Squares (OLS) multiple regression analyzes were used to identify the predictors that contribute to explaining the four dependent variables. Although OLS analyses are more likely to present the challenge of uncontrolled biases, it has been observed that other approaches only outperform OLS in larger samples (DeMaris 2014). Given that our sample is quite small, we found OLS an appropriate approach for the regression analysis. Each mobility dimension was regressed on the eight control variables in the first step and the seven personal dispositions in the second step. To this end, additional regression analyzes were computed, with the product terms of the seven personal dispositions and the dummy variables for country being entered in the third and final steps to analyze potential cross-cultural differences of the impact of the personal dispositions. Note that as none of the interaction terms became significant, the results of the first two steps only are documented in Tables 4 and 5. Overall, a total of 30 percent of the variance in general geographic mobility readiness (*F* = 5.50; *p* < 0.001), 24 percent of the variance in geographic mobility readiness for career incentives (*F* = 4.32; *p* < 0.001), 21 percent of the variance in geographic mobility readiness for social incentives (*F* = 3.57; *p* < 0.001), and 19 percent of the variance in geographic mobility readiness for material incentives (*F* = 3.17; *p* < 0.001) was explained by our regression equations.

**Table 4:** Explaining general geographic mobility readiness and geographic mobility readiness for various incentives

		General Geographic Mobility Readiness					Mobility Readiness for Career Incentives				
		*B*	SE *B*	Lower CI	Upper CI	β	*B*	SE *B*	Lower CI	Upper CI	β
Step 1	Country of origin	0.02	.19	− 0.35	0.39	.01	− 0.27	.21	− 0.68	0.14	− .15
	Gender	0.03	.12	− 0.21	0.27	.02	0.07	.13	− 0.20	0.33	.04
	Age	0.03	.03	− 0.06	0.07	.01	0.03	.04	− 0.04	0.11	.08
	Relationship status	0.08	.11	− 0.13	0.29	.05	0.12	.12	− 0.11	0.36	.07
	Expected graduation date	0.00	.15	− 0.30	0.30	.00	− 0.04	.17	− 0.37	0.30	− .02
	Job in university town	− 0.14	.04	− 0.22	− 0.06	− .24***	0.01	.05	− 0.08	0.10	.02
	Number of prior relocations	0.06	.09	− 0.11	0.23	.06	− 0.14	.10	− 0.34	0.05	− .13
	Distances of prior relocations	0.11	.07	− 0.03	0.25	.14	0.13	.08	− 0.03	0.28	.15
	Step 1: Δ*R*^2^	.17	***				.05				
Step 2	Uncertainty tolerance	0.23	.08	0.08	0.38	.18**	0.07	.09	− 0.20	0.24	.05
	Horizontal individualism	− 0.10	.08	− 0.26	0.06	− .08	0.01	.09	− 0.17	0.19	.01
	Vertical individualism	0.06	.08	− 0.09	0.22	.06	0.42	.09	0.25	0.59	.36***
	Horizontal collectivism	− 0.13	.11	− 0.35	0.08	− .09	− 0.08	.12	− 0.32	0.15	− .05
	Vertical collectivism	0.00	.08	− 0.15	0.15	.00	− 0.01	.08	− 0.18	0.16	− .01
	Parents’ attitude to GM	0.12	.06	0.01	0.24	.14*	0.21	.07	0.08	0.34	.23**
	Peers’ attitude to GM	0.23	.07	0.10	0.37	.23**	0.13	.08	− 0.02	0.28	.13
	Constant	2.53					0.99				
	Step 2: Δ*R*^2^	.13	***				.19	***			
	*R*^2^	.30	***				.24	***			

For country of origin, 0 = Spain; 1 = Germany. For gender, 0 = male; 1 = female. For relationship status, 0 = single; 1 = in relationship. For expected graduation date, 0 = next year; 1 = in two years or later. The probability of finding a job in the university town after graduation ranged from 1 “very improbable” to 6 “very probable”. Distances of prior relocations varied from 0 = “no relocation,” to 3 = “relocation to another country”. For all other scales, high values represent a strong endorsement of the construct. CI = confidence interval

*p < .05; ** *p* < .01; *** < .001 (two tailed tests)

**Table 5:** Explaining general geographic mobility readiness and geographic mobility readiness for various incentives

		Mobility Readiness for Social Incentives					Mobility Readiness for Material Incentives				
		*B*	SE *B*	Lower CI	Upper CI	β	*B*	SE *B*	Lower CI	Upper CI	β
Step 1	Country of origin	− 0.58	.23	− 1.04	− 0.13	− .29*	− 0.62	.27	− 1.14	− 0.09	− .27*
	Gender	− .0.05	.15	− 0.35	0.25	− .02	0.25	.17	− 0.09	0.60	.11
	Age	− 0.03	.04	− 0.11	0.06	− .05	0.02	.05	− 0.07	0.11	.04
	Relationship status	0.12	.13	− 0.14	0.38	.06	0.17	.15	− 0.13	0.47	.08
	Expected graduation date	− 0.32	.19	− 0.70	0.05	− .15	− 0.30	.22	− 0.73	0.14	− .12
	Job in university town	0.02	.05	− 0.08	0.12	.03	0.18	.06	0.06	0.29	.22**
	Number of prior relocations	− 0.02	.11	− 0.23	0.20	− .01	− 0.03	.13	− 0.28	0.22	− .02
	Distances of prior relocations	0.02	.09	− 0.15	0.19	.02	0.08	.10	− 0.12	0.27	.07
	Step 1: Δ*R*^2^	.13***					.12**				
Step 2	Uncertainty tolerance	0.08	.10	− 0.12	0.26	.05	0.07	.11	− 0.14	0.29	.04
	Horizontal individualism	− 0.02	.10	− 0.22	0.18	− .01	0.11	.12	− 0.11	0.35	.07
	Vertical individualism	− 0.06	.10	− 0.25	0.13	− .05	0.09	.11	− 0.13	0.30	.06
	Horizontal collectivism	0.41	.14	0.15	0.68	.23**	− 0.43	.16	− 0.74	− 0.12	− .21**
	Vertical collectivism	0.12	..10	− 0.07	0.30	.09	0.21	.11	− 0.01	0.43	.15
	Parents’ attitude to GM	0.12	.07	− 0.02	0.27	.12	0.21	.09	0.04	0.38	.18*
	Peers’ attitude to GM	− 0.04	.08	− 0.20	0.13	− .03	− 0.05	.10	− 0.24	0.14	− .04
	Constant	2.17					2.10				
	Step 2: Δ*R*^2^	.08**					.07*				
	*R*^2^	.21***					.19***				

For country of origin, 0 = Spain; 1 = Germany. For gender, 0 = male; 1 = female. For relationship status, 0 = single; 1 = in relationship. For expected graduation date, 0 = next year; 1 = in two years or later. The probability of finding a job in the university town after graduation ranged from 1 “very improbable” to 6 “very probable”. Distances of prior relocations varied from 0 = “no relocation,” to 3 = “relocation to another country”. For all other scales, high values represent a strong endorsement of the construct. CI = confidence interval

*p < .05; ** *p* < .01; *** < .001 (two tailed tests)

Besides the probability of finding a job in the university town, *general geographic mobility readiness* was predicted by parents’ and peers’ perceived attitudes to geographic mobility and by uncertainty tolerance – thus confirming our first and third hypotheses. The less likely the business management students thought they were to find a job in Berlin or Madrid, the more strongly they perceived their parents and peers to be in favor of geographic mobility, and the more uncertainty tolerant they were, the stronger their general geographic mobility readiness. As personal dispositions were also considered, the mean difference found in the ANOVA for country of origin itself was no longer significant.

*Geographic mobility readiness for career incentives* was predicted by vertical individualism, as well as by parents’ perceived attitudes to geographic mobility, thus confirming our second and third hypotheses. The higher the students scored on vertical individualism and the more positive they perceived their parents’ attitudes to mobility as being, the more willing they were to move if certain career incentives were offered.

*Geographic mobility readiness for social incentives* was associated with the country of origin and horizontal collectivism. Besides the country effect described above, the higher the students scored on horizontal collectivism, the more willing they were to be geographically mobile when social incentives were offered.

The probability of finding a job in the university town, parents’ perceived attitudes to geographic mobility, and horizontal collectivism predicted *geographic mobility readiness for material incentives*, again confirming our second and third hypotheses. The higher the perceived likelihood of students finding a job in Berlin or Madrid, the more positive they perceived their parents’ attitudes to mobility, and the higher their horizontal collectivism scores, the more willing they were to move for material reasons. Note that the probability of finding a job in the university town after graduation was positively associated with the dependent variable. This is in line with the reasoning that people who think it is possible to get a job in their place of residence are only willing to move only when offered material incentives.

## Discussion

### Cultural differences

The study examined the willingness to be geographically mobile from a cross-cultural perspective, particularly among German and Spanish undergraduate business management students. To our understanding, there are not many research findings explaining geographic mobility readiness using this perspective. We found significant differences in individualism and collectivism, with the exception of horizontal individualism, which did not differ between the groups. The German business management students scored higher on vertical individualism *(H4b)* and lower on horizontal and vertical collectivism *(H4c)* than the Spanish students. No significant differences in uncertainty tolerance *(H4a)* were found, however. Note that we used the personal disposition uncertainty tolerance measure (Dalbert 2002) to reflect Hofstede’s cultural dimension of uncertainty avoidance in the present study. Apart from the fact that our method and sample differ from Hofstede’s, it is also possible that our concept of uncertainty tolerance is not close enough to Hofstede’s. Another explanation would be that globalization has increased the acceptance of uncertainty in Spanish culture and thus diminished differences between the cultures.

One could query whether cultural differences between Germany and Spain remain the same in Hofstede’s research. Yet, research brought to light that Spain has changed to a more individualistic country, and some studies have indicated Spain to be more individualistic than Germany or even the USA (Spector et al. 2001). Moreover, more current findings suggest, for example, Germany to be less individualistic than various Latin American countries (i.e. Chile, Mexico, and Venezuela), which is contradictory to Hofstede’s (1980) findings. Unfortunately, however, in those studies, Spain was not investigated (Fernandez et al. 1997; Merritt 2000). This is also true for uncertainty avoidance with studies showing Spanish people to be less uncertainty avoidant than Germans or any other studied culture (Spector et al. 2001). As Hofstede’s data are more than 30 years old it seems to be reasonable that the cultures of some nations have changed given the globalization influence. This is echoed by Fernandez that “although a nation’s values are deep-seated preferences for certain end states, they are subject to change over the years as external environmental changes shape a society” (Fernandez et al. 1997, p. 52).

The expected cultural differences between the German and the Spanish business management students in terms of the general geographic mobility readiness *(H5)* were confirmed, with the German students being generally more willing to relocate. Considering the specific facets of readiness to move, Spanish students were more willing to be mobile when offered social incentives. Moreover, Spanish business management students were also more willing to move than their German counterparts when offered material incentives, but this difference could be traced back to the subjective probability of finding a job in the university town (Baluku et al. 2018). As the Spanish students were more confident about finding a job in Madrid, material incentives were needed to increase their geographic mobility readiness. In sum, whereas the Spanish students were, in general, less willing to relocate than their German counterparts, they were more receptive to mobility when incentives are offered.

### Understanding geographic mobility readiness

Comparison of the various predictors in our regression analyzes showed that the parents’ perceived attitudes to geographic mobility were particularly important *(H3a)*. This was the only variable that was able to predict three of the four dependent variables (notice that the relationship with the fourth variable was marginally significant). This result unambiguously illustrates the major role of subjective norms in explaining behavioral intentions (Ajzen 1991). These findings are in line with the work of Dette and Dalbert (2005), who investigated German students shortly before graduating from high school. Whereas these authors found higher correlations for friends’ attitudes than for parents’ attitudes to mobility, however, in the present study, parents’ perceived attitudes to geographic mobility proved to be even more salient than peers’ perceived attitudes *(H3b)*. Our results indicate that parents are important sources of advice and support on job-related decisions (Harris 1995) and are in line with studies showing that parents play a major role in their children’s occupational decision-making processes (Kracke 1997; Lee et al. 2019; J. Liu et al. 2015; Y. Liu et al. 2020; Whiston and Keller 2004). This also supports previous research that has identified family related antecedents of mobility decisions (Gripenberg et al. 2013; Mutter 2017; Patton and Doherty 2017; Tarique and Weisbord 2013).

Uncertainty tolerance predicted general geographic mobility readiness in the expected direction *(H1)*. The higher the business management students were in uncertainty tolerance, the more willing they were to move for job-related reasons. The same did not hold when incentives were offered, however. A second difference between general and incentive-related mobility readiness was that only the latter was associated with individualism-collectivism *(H2a and H2b)*. This pattern of results seems to indicate a somewhat different dynamic concerning the two kinds of geographic mobility readiness. Whereas personality, as reflected by uncertainty tolerance, may explain the general mobility readiness, the importance of social integration, as reflected on individualism-collectivism, seems to affect incentive-related mobility readiness only. More collectivist students value their social integration and may believe that leaving the group will have negative consequences for them, which they may consider as a high price to pay for geographic mobility. Thus, the more collectivistic the students were, the more important incentives to compensate for the social costs of relocation became. This particularly manifested in the Spanish students. Finally, in line with Dette and Dalbert (2005), vertical individualism was positively associated with career-related mobility. Business management students who score high in vertical individualism believe that the individual is autonomous and thus free to move. They also compete with others and focus on status differences and hierarchy in their efforts to get on the top. They are thus most likely to be attracted by career incentives. The differences in the effects of collectivism and individualism on mobility may further be explained by flexibility. Flexibility is an important predictor of career mobility (Baluku et al. 2018), yet flexibility tends to be high in individualistic societies (Cheng et al. 2014).

Besides the country of origin, the probability of finding a job in one’s university town was the only one of the eight control variables to reveal significant differences in geographic mobility readiness. Spanish students were more optimistic about finding a job in Madrid and, consequently, generally less willing to move than German students. The prospect of material incentives increased the willingness of those expecting to find a job in their university town to move after graduation. Prior relocations, a predictor found to be important in several previous studies (Froese et al. 2013; Landau et al. 1992; Stumpf 2014), did not yield significant results. One explanation might be that earlier relocations had been decided by the parents, and might not have any impact on the students’ geographical mobility. However, Myers (1999) shows that the frequency of moving in childhood predicts the probability of moving in adulthood.

### Limitations and suggestions for future studies

The present study has several shortcomings that should be taken into consideration when applying the findings. First, the study was cross-sectional in nature. Moreover, the sample was composed of highly qualified students that come from the business management discipline, which is characterized by being “global” and having more flexible job offers in terms of location. Our sample is also drawn from arguably leading universities in the respective countries, yet these universities are located in big and metropolitan cities, therefore expected to have better employability. Consequently, the sample is not comparable to the rest of the Spanish and German student population or labor force. Although we went to great efforts to recruit comparable samples in the two cultures, it cannot be ruled out that the differences observed are not cultural, but sample-specific differences.

Second, we applied Ordinary Least-Squares (OLS) approach in our regression analyses. This approach has been critiqued as inadequate and likely to involve effects of uncontrolled biases (Ugrinowitsch et al. 2004), such challenges are less likely when the sample is small or when a higher order variable is added to the regression analysis (DeMaris 2014; Huang 2018). Moreover, although we find statistically significant associations, the size of the effects in some instances are small. Hence replication studies are necessary if our findings are to be applied with confidence.

Third, there might be some background variables essential in shaping geographic mobility readiness and decisions that we were not able to control for as the socio-economic status of the students. Thus, this study should only be seen as a first attempt to analyze geographic mobility across cultures. Additionally, our study focuses on two European countries. Since the European Union has a unified labor market, there are fewer barriers to career-related geographical mobility such as language and country-specific employment requirements. However, there are several constraints to mobility between developing and developed countries and between continents. It could therefore be interesting for future research to explore differences in geographical mobility readiness among countries with differing economic and development realities. Nonetheless, even in Europe, countries responded differently to the recent economic crisis. Some countries such as Spain, Greece, Croatia, and Italy (compared to Germany) experienced high unemployment in the aftermath of the economic crisis (Verd et al. 2019) suggesting that some labor markets are more broken than others. Yet the structure of the labor market could have strong effects on young people’s geographical mobility readiness. Hence the need for studies comparing a wide range of countries in Europe and beyond, as well as considering the impact of labor market structure on the association between cultural orientations and career-related geographical mobility readiness.

Forth, we investigated geographic mobility readiness, but not mobility behavior. Although studies have found a relationship between the two (Brett and Reilly 1988), the impact of willingness to move on actual job transfer decisions is unclear. The construct investigated here is more specific than an attitude, but less closely related to real behavior than an intention (Ajzen 1991). Future studies should explore behavioral intentions and actual behavior by investigating the career decisions and mobility behavior of students nearing the end of their studies and taking up their first job after graduation.

### Practical and theoretical implications

The study offers important insights that could be useful to organizations seeking to recruit mobile young professionals. Our results show that prospecting graduates are less ready for job-related geographic mobility when chances of finding a job in the home town are high. However, they tend to be more ready when material incentives are attached. This indicates that organizations seeking to attract young talented professionals into jobs that require geographical relocation or frequent international travel should provide attractive incentives for such jobs. It is rather logical that an individual will relocate for a job if the pay (Tekleselassie and Villarreal 2011) and related benefits are higher than those offered for jobs in one’s hometown or country. However, social incentives are also appealing for individuals from more collectivistic societies.

In a highly globalized economy, companies are no longer confined to locations where they have been founded. Many organizations are now internationalized; short-term or long-term international assignments are inevitable. Hence, it is essential that graduates seeking work in organizations today be ready for geographical mobility. Career counselors and educators should acknowledge this reality and work towards improving mobility attitudes of prospecting graduates. Our findings reveal that vertical individualism is related to readiness for career-related geographical mobility; thus highlighting the need for freedom of thought and decision-making. Career counselors and educators, therefore, need to foster the development of autonomy among prospecting graduates. Not only may it be necessary for deciding to pursue a career abroad, but also generally important for autonomous career decisions that may foster speedy school-to-work transition. Moreover, the perception of parents’ attitudes towards job-related geographical mobility is emphasized. If autonomous decision-making is developed during schooling, the impediment of perceived negative parental attitudes may be overcome. However, interventions for both students and parents geared towards appreciating and embracing the protean nature of today’s careers may be useful.

The field of career expatriation is a growing area of research. However, we found no studies focusing on career-related geographic mobility readiness at a cross-cultural level; and more specifically there are no studies analyzing the geographical mobility readiness of German and Spanish university students. This is among the first efforts in this direction. Therefore, further comparisons between different countries are needed to broaden the perspective on mobility. Additionally, an interdisciplinary framework covering political, social, economic, and cultural aspects should be applied. The individualism-collectivism construct should be included in future cross-cultural research on mobility, as it proved to be a good predictor of the willingness to relocate; moreover, it is a universal dimension for cultural research. Uncertainty tolerance has emerged as an important predictor of mobility readiness in several studies and across different mobility dimensions (Dette and Dalbert 2005; Kathleen Otto et al. 2010), but the expected cultural differences were not confirmed in the present study. The relationship between uncertainty avoidance and uncertainty tolerance, and the implications of the two for mobility, thus warrant further investigation. Based on a German-Canadian comparison study it seems that both concepts are not highly correlated (Kathleen Otto et al. 2011). Broadening the geographical scope of the study to include more countries across the different continents might be useful. Subjective norms and, in particular, parents’ attitudes to mobility should certainly be included in future research on adolescents’ and young adults’ mobility readiness, as their perceived attitudes significantly predicted all kinds of mobility investigated here.

## Conclusion

The present study used a multi-method approach (Campbell and Fiske 1959) to investigate business management students’ mobility readiness for their first job after graduation. Overall, the results were in line with our hypotheses. Cultural differences emerged between the German and the Spanish students. Specifically, German students score higher on vertical individualism and lower on vertical and horizontal collectivism than their Spanish counterparts. Consequently, German students were generally more willing to be geographically mobile. However, the willingness for geographic mobility was higher when social or material incentives are offered. General geographic mobility readiness was predicted by parents’ and peers’ perceived attitude towards mobility; as well as by uncertainty tolerance. Geographic mobility readiness for career incentives and for social incentives were predicted by vertical individualism and horizontal collectivism, respectively.

Career mobility in general and geographic mobility, in particular, are becoming increasingly important in times of globalization. This applies to the majority of cultures, independent of their mobility traditions and cultural orientations. From an employees’ perspective, the readiness to move may be the decisive factor in a successful career. From the company’s point of view, it is becoming more important to understand employees’ motivations and to offer the right incentives. We believe that our study serves as a good starting point by demonstrating two ways of assessing different kinds of geographic mobility readiness and identifying three important sets of predictors. Our findings are in line with the idea that the value attached to social networks, the tolerance of uncertainty, and the attitudes of the social environment to mobility shape the willingness to relocate. Finally, our study supports the assumption that not only do people in different cultures show differential willingness to move but also that the impact of incentives may differ across cultures. Despite these interesting findings, our study is limited by the self-report measures and the cross-sectional approach. Moreover, our sample is drawn from population management students in two major cities in Europe. Hence not representative of the general student population or representative of the young people in the labor market. Longitudinal cross-cultural studies could be useful in explaining how these antecedents affect geographic mobility willingness/ readiness over time and consequently actual movement/ expatriation in different cultural and economic contexts.

## Supplementary Information

Below is the link to the electronic supplementary material.

Supplementary file1 (DOC 33 kb)

## Data Availability

Data used for this study can be obtained from the corresponding author upon a written request.

## Footnotes

1 According to country comparisons on https://www.hofstede-insights.com/product/compare-countries/, Germany has a higher than Spain on individualism.

2 Data available on https://www.hofstede-insights.com/product/compare-countries/ show that Spain has higher uncertainty avoidance score than Germany.
