# Supplementary material for: Cultural differences in geographic mobility readiness among business management students in Germany and Spain ahead of graduation
Source: SN Soc Sci. 2021 Jun 25;1(7):161. doi: 10.1007/s43545-021-00171-0 (PMC8225400; doi:10.1007/s43545-021-00171-0)
Supplement: Supplementary file 1 — Supplementary file1 (DOC 33 kb) [file 43545_2021_171_MOESM1_ESM.doc]

###### **Appendix**

*Items of the “Mobility for Incentives” Scale*

Imagine you are looking for a job. You have contacted several companies and received two positive replies. Now you have to choose between the job offers:

*Situation 1:* Imagine you have two good job offers. One is in your university town; the other is located so far away that you would have to move to a new city within your country. How likely would you be to accept the offer of the job in the other city?

I would accept the offer if...

(1.) … I earned more money there.

(2.) … I worked with nice people.

(3.) … I had better career prospects.

(4.) … I had a company car.

(5.) … I already knew people there.

(6.) … it meant professional development.

(7.) … the company provided me with low-rent housing.

(8.) … I had relatives there.

(9.) … I would have a higher position there.

*Situation 2*: Imagine you have two good job offers. One is in your university town; the other involves being away from home for several weeks at a time. How likely would you be to accept the offer with changing locations?

I would accept the offer if...

(see, above for the single items)

*Situation 3*: Imagine you have two good job offers. One is in your university town; the other is for an international firm and requires that you work in another European country for the first six months. How likely would you be to accept the offer involving a move abroad?

I would accept the offer if...

(see, above for the single items)
